# Dispersion State and Damage of Carbon Nanotubes and Carbon Nanofibers by Ultrasonic Dispersion: A Review

**DOI:** 10.3390/nano11061469

**Published:** 2021-06-01

**Authors:** Harald Rennhofer, Benjamin Zanghellini

**Affiliations:** Department of Materials Science and Process Engineering, Institute of Physics and Materials Science, University of Natural Resources and Life Sciences, Vienna, Peter-Jordan-Strasse 82, A-1190 Vienna, Austria; harald.rennhofer@boku.ac.at

**Keywords:** ultrasonication, dispersion, carbon nanotubes, CNT, damage

## Abstract

Dispersion of carbon nanotubes and carbon nanofibers is a crucial processing step in the production of polymer-based nanocomposites and poses a great challenge due to the tendency of these nanofillers to agglomerate. Besides the well-established three-roll mill, the ultrasonic dispersion process is one of the most often used methods. It is fast, easy to implement, and obtains considerably good results. Nevertheless, damage to the nanofibers due to cavitation may lead to shortening and changes in the surface of the nanofillers. The proper application of the sonicator to limit damage and at the same time enable high dispersion quality needs dedicated knowledge of the damage mechanisms and characterization methods for monitoring nano-particles during and after sonication. This study gives an overview of these methods and indicates parameters to be considered in this respect. Sonication energy rather than sonication time is a key factor to control shortening. It seems likely that lower powers that are induced by a broader tip or plate sonicators at a longer running time would allow for proper dispersions, while minimizing damage.

## 1. Introduction

Carbon-fiber-reinforced polymers (CFRP) are advanced light-weight materials for high-end applications like aeronautics or the automotive industry [1,2,3]. With regard to fracture, the materials’ properties are outstanding, but can even be improved by the application of polymer resin modified by nano-particles [4]. Different nano-fillers are used in research and industry, but most often ceramics and carbon-based filler materials are applied [5,6,7]. The latter would be graphene, carbon nanotubes (CNT)—single walled or multi walled (SWCNT/MWCNT)—and carbon nano-fibers (CNF). CNT are about 1–100 nm in diameter and up to several µm in length, while CNF are about 100 nm in diameter and up to several µm in length, always depending on the specific fiber type [8,9]. In this publication, we mainly treat the dispersion of CNT. Not only CFRP, but also the polymer alone might show enhanced fracture characteristics due to application of nanofillers in the matrix [10,11]. In general, this is due to the influence that nanofillers have on the crack initiation and crack propagation in the composite. Fibrous fillers with a high aspect ratio allow, for example, crack bridging or fiber pull-out, depending on the interaction with the resin [12,13,14].

There are two crucial process steps for the successful implementation of nano-particles into a polymer matrix: functionalization and dispersion. Both help to avoid agglomerations of nano-fillers, which would inevitably weaken the composite. A possible functionalization of the nano-filler enhances separability of the nano-particles and, if applicable, also allows cross-linking to the polymer itself (e.g., by amid bonds). This is usually applied to CNT and achieved by chemical treatment by, for example, NaNO_3_/H_2_SO_4_/KMnO4 [15], HNO_3_/H_2_SO_4_ [16], or H_2_O_2_ [17]. The type and amount of functionalization controls the nanofiller–matrix interface, and thus directly affects the failure mechanisms. It therefore allows the optimization of the materials’ properties, for example, to obtain either high strength or high toughness [18,19]. Dispersion—that is, the proper distribution of nano-fillers in the matrix—must be realized and further maintained throughout the full production process. This is achieved by several methods [20,21]; for example, it can be performed in an optimal way by a calender [22]. In the calender, the shear forces to break the nano-filler agglomerates arise from transporting the resin with the nano-fillers through the small gap of two cylinders, rotating in opposite directions. Gap size and rotation speed influence the dispersion quality. Usually, several dispersion runs with a stepwise-reduced gap size are used [22,23], resulting in fine dispersed nano-fillers and very small size of remaining agglomerates. Calendering is nevertheless time consuming and not easily adapted to a high throughput or being scaled up, as would be advantageous in industrial application.

The calender can, in principle, be replaced by ultrasound dispersion, which is by now a widely used method to disperse nanofillers in solution [24,25,26,27]. The biggest difference in the application of both methods is that with the calender nanofillers are dispersed directly in the resin, while ultrasound nanofillers are most often dispersed in an additional solvent first [28,29,30]. This solvent is later mixed with the resin and subsequently evaporated. The main reason for this additional step is the high viscosity of most resins, which does not easily allow direct dispersion by ultrasound. Only a few publications report on dispersion with ultrasound directly in the undissolved resin or hardener [17,24,31,32,33]. This is usually possible for low-viscosity systems. Independent of the matrix, the most important parameters reported for high dispersion quality are the ultrasonication time and the applied power per volume [34,35]. In the ultrasound method, those shear forces acting to break up agglomerates mainly arise from cavitation phenomena. These local pressure peaks can additionally lead to damage of the nano-fillers, changing surface properties or even shortening fibrous nano-fillers [36,37,38]. This would directly influence the properties of the produced polymer composites or CFRP. Thus, it is crucial to understand the effects that ultrasonication has on the nanofillers’ properties. Although there are several publications reporting on such effects [17,34,39,40,41], to the knowledge of the authors, there is no concise overview of this literature. With this paper, we aim at giving an overview of effects that ultrasonication has on the properties of elongated nano-fillers like CNT and CNF. We want to limit this review to the discussion of the nano-fillers and will not address changes in nano-composites’ properties.

We present a short introduction to standard dispersion methods, followed by the consideration of ultrasound dispersion. We present the basic concept of cavitation, damage mechanisms during ultrasonication treatment, and methods to estimate damage; measure the dispersion state; and give an overview of relevant publications that specifically investigated damage to CNT by sonication. After a short discussion of ultrasound dispersion in the view of the reported publications, we conclude the manuscript.

## 2. Dispersion

### 2.1. Overview of Dispersion Methods

#### 2.1.1. Short Overview of Dispersion Methods Other Than Ultrasonication

In the literature, several methods for dispersion of nanofillers into resin are described. Among others, there are the following: calender [22,42], ball mill [20,43,44,45,46,47], high shear mixing, extrusion [32,48,49,50], high speed flow and high pressure jet mill [21], and ultrasound [24,25,26]. In all of these methods, agglomerates are broken up by either direct mechanical force (e.g., with cylinders in the calender or beads in the ball mill), turbulent flow (e.g., in the high-pressure jet-mill), or cavitation during sonication. Each method has its benefits and limitations, which will not be discussed here. An overview and discussion of these methods can be found in [20,21,51].

#### 2.1.2. Ultrasonication—Basic Considerations

**Figure 1:**
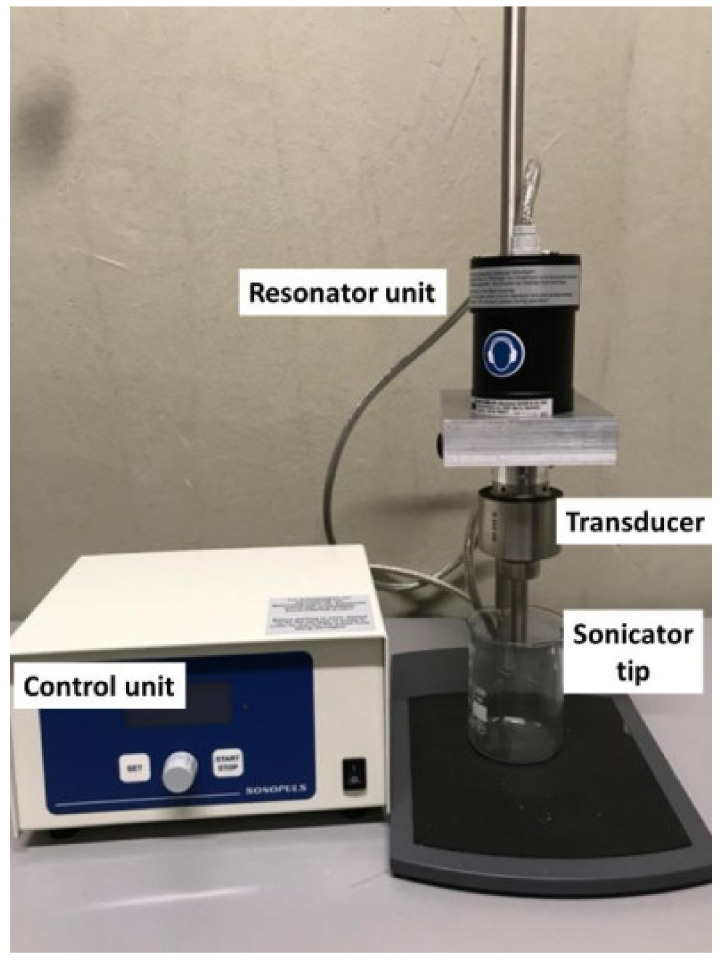
Example of sonication equipment with the main parts indicated.

In a standard setup, ultrasound is generated by an ultrasound resonator and transduced by a connecting part, the transducer, which is in resonance with the ultrasound generator, into the liquid. For most laboratory systems applied for dispersion, this is a rod that narrows towards the liquid. Thus, a much higher power density can be applied with respect to a standard sonication bath. An example of such a standard laboratory ultrasound dispersing setup is depicted in Figure 1. The sound waves induce a compression and decompression front moving through the liquid that interacts with the liquid molecules and small gas bubbles in the liquid. These pressure fluctuations lead to growth and subsequently collapse of gas bubbles, which is called acoustic cavitation. The energy required to generate bubbles, the mean size of bubbles and the effect of the cavitation on the surrounding media depends on many factors, such as viscosity and density of the media and frequency of the ultrasound. The bubbles usually grow into the micrometer size regime and collapse in fractions of microseconds. This leads to a drastic increase in pressure and temperature, affecting the surrounding media in several ways, such as generation of radicals like OH, sonoluminescence (light emission), and shock waves that act as shear forces in the surrounding liquid [52,53]. The shape of the ultrasound transducer (rod-like horn or plate-type transducer) influences the energy density, and thus can influence the effect caused in the media. The standard ultrasonic bath, in particular, has a much lower power density with respect to a horn sonicator. Most publications presented in this review actually applied horn sonicators. Under proper conditions, standing waves can form in the liquid, which change the type and distribution of bubbles, and thus affect the liquid. In general, one distinguishes two types of bubbles: bubbles that oscillate in size over a longer period of time, which are called “stable”, and bubbles that collapse rather fast, which are called “transient” or sometimes also “inertial”. At lower ultrasonic energy densities, standing waves are more likely to form, and the proportion of stable bubbles predominates. At higher energy densities, chaotic cavitation phenomena with transient bubbles and fewer or no standing waves dominate [54]. The use of probe sonicators was reported to sometimes contaminate the sample by small particles from the sonication tip resulting from tip erosion. This could be avoided by the use of a vial sonicator [26].

The shear resulting from the cavitation-induced pressure front is frequently used to break up agglomerates, that is, for dispersion of CNT and CNF (e.g., [38]) and dispersion of other nano-particles in solution (e.g., [55]). This is achieved by disentanglement of single fibers from the agglomerates. The ultrasound dispersion has been used in various ways. For studies concerning the effect of sonication on the nano-fillers, direct dispersion in a solvent system is usually applied (e.g., [25,38,56]). For the production of composites, CNT are also most often first suspended in a solvent system by sonication. Thus, results can readily be linked. The solvent system itself depends on the type of composite, for example, thermoplast or epoxy system. The nano-filler suspension is then mixed with the polymer and evaporated afterwards [57,58,59,60], which may be followed by further process steps, for example to remove trapped air, before curing. Only a few publications deal with dispersion of CNT directly in resin or hardener without additional solvent [17,24,31,32,33]. This is only possible for low-viscosity systems. Cement composites are produced in a similar way as the polymer systems: first producing a suspension of CNT in, for example, water, with the addition of superplasticizer by sonication, and then high-speed shear mixing of this suspension with cement powder [40,41]. Often, publications dealing with the effect of sonication do not address damage on CNT, but directly investigate products’ properties, such as mechanical properties, electrical conductivity, or thermal properties of various composites [10,34,39,40,41,61].

### 2.2. Effects of Ultrasonication

#### 2.2.1. Damage Mechanism

While using ultrasonication for the break-up of agglomerates, one should bear in mind that the energy deposited in the liquid and the resulting cavitation does not only show the wanted effect of dispersion of nano-particles, but can also induce other processes like sonochemistry (e.g., generation of radicals [52]), directly damage the nano-particles [36,37,38,62], or even damage the sonicator probe of the ultrasonic equipment [55,63]. Therefore, it is important to consider damage mechanisms that might affect the nano-particles. In the following, the possible damage mechanisms reported in literature are presented.

**Figure 2:**
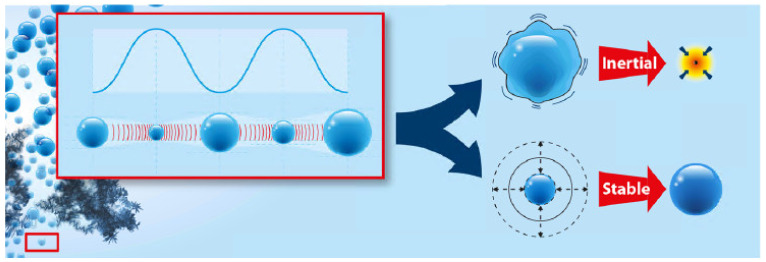
Display of the two possible cavitation modes, reprinted with permission from [38], Copyright (2013) American Chemical Society. The bubble size oscillation with ultrasonic pressure waves is indicated in the left part of the scheme. Depending on the power, this leads to transient (inertial) cavitation, that is, instable bubbles that collapse after a few oscillation cycles, or stable bubbles that oscillate in size over many more ultrasonic wave cycles.

The damage of nano-particles during ultrasonic treatment is reported to depend on different factors: on the physical properties of the nano-particle, including dimensions (i.e., length and diameter) [37]; on the liquid into which the particles are dispersed; and mainly, on power (density), and in a way, on sonication time. The power does change the cavitation type, and thus has a direct influence on the shock wave in the liquid and on the forces acting on the nano-particles. Sesis et al. reported on the different effect of the two cavitation types, namely “stable” (formed at lower power) and “transient” (also “inertial”, formed at higher power), that lead to different types of damage of single-walled carbon nanotubes. The two cavitation types are depicted in Figure 2. In this context, the formation of H_2_O_2_ was proposed as a measure for stable cavitation, and the amount of a MHz broadband noise, as a result of the bubble collapse, was proposed as a measure of the amount of transient cavitation, respectively. It was also stated that neither the input power nor the heat transfer to the solution by ultrasonication can be used as direct measures for the amount of cavitation, but sonication energy is a suitable parameter. Stable cavitation (lower power) leads mainly to surface damage, while transient cavitation (higher power) results in exfoliation and shortening of CNTs [38].

Length reduction of CNT is caused by two main effects: one is bending and buckling of the CNT, the other stretching and rupture. Both effects are reported to start immediately with sonication. The length *L* of the CNT in both cases is reported to decrease with a power law dependence on the sonication time *t: L(t)~t^−m^* with different values for the exponent *m*. Pagani et al. showed by modelling that shortening caused by buckling results in a power law exponent *m* of about 0.205–0.250, while scission due to stress along the CNT results in a power law exponent *m* of about 0.41–0.50. It is argued that longer fibers tend to be oriented tangentially next to expanding and subsequently collapsing bubbles, while shorter fibers are more easily rotated and are oriented radially in this process. The liquid flow caused by the cavitation bubble collapse is faster close to the bubble surface, resulting in a drag on the center of tangentially aligned CNT, leading to buckling of longer fibers (*m* = 0.205–0.250). The same effect shows higher flow speed at the tip of a radially oriented fiber, resulting in stretching of shorter fibers (*m* = 0.41–0.50).

**Figure 3:**
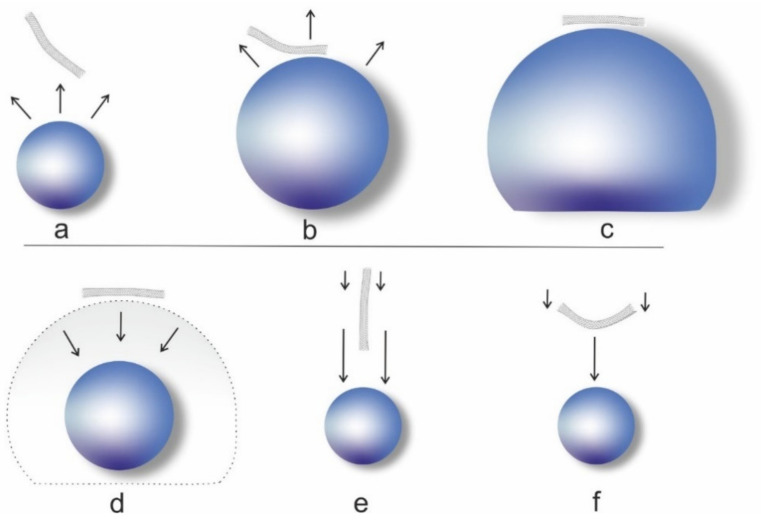
Effect of the cavitation bubble on the CNT, authors’ drawing inspired by [62]: (**a**) bubble growth in the vicinity of a CNT; (**b**) bubble begins to push and reorient CNT; (**c**) the CNT is oriented tangentially; (**d**) collapse of the cavitation bubble leads to a drag in the moving liquid that is faster in the center and slower towards the ends of the CNT, which leads to either (**e**) radial orientation with faster flow at the tip and slower flow at the end, which stresses the CNT, or (**f**) bending of the CNT in the center and subsequently buckling.

Over a longer sonication time, the CNT would become shorter and the dominant mode of failure would thus also change from buckling to stretching, with an according change in the scission rate. Which mode is dominant at the beginning and the critical length at which the mode changes depends on dimensions (diameter, wall thickness, length), Young’s modulus, and stiffness of the CNT and would likely be different for SWCNT and MWCNT [62]. The principle of the two shortening mechanisms is depicted in Figure 3.

Shortening of SWCNT following a power law with exponent *m* = 0.49 was reported, for example, by Hennrich et al. SWCNT were length-selected by size exclusion chromatography, and a fraction with a mean size of about (800 +/− 300) nm long SWCNT was shortened by sonication until a mean length of about (165 +/− 80) nm was reached after 120 min. The length of the nanotubes is not only found to decrease, but the length distribution narrows down, an effect that is caused by a reduced number of longer CNT in the sample. It was demonstrated that the following shortening mechanism is likely: cavitation bubbles are imploding, resulting in movement of liquid. The liquid is acting like friction along the CNT, which is stressing the CNT until its tensile strength is exceeded and the CNT is ripped apart in the middle. The similarity to the process described by [62] is stated. This ripping or radially oriented CNT is also depicted in Figure 3e. From the data, Hennrich et al. conclude that shortening continues until most tubes are reduced to a lower length limit, at which point the friction forces are too small to further shorten the CNT. After that, no considerable reduction in CNT length with longer sonication times would be observed [36].

**Figure 4:**
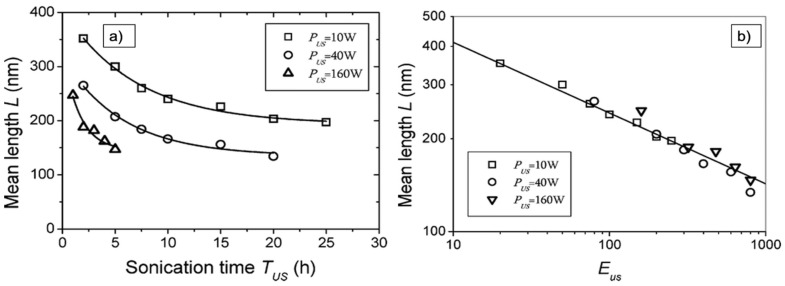
Display of the power law behavior of the mean length of MWCNT shortened by sonication. (**a**) Length as a function of the sonication time; (**b**) length as a function of the sonication energy, exhibiting L~E-0.21 for all applied sonication powers. Reprinted with permission from [37], Copyright (2009) American Chemical Society.

Lucas et al. argue that scission would continue at a smaller and smaller rate until a terminal length of well below 100 nm was reached, depending on the stiffness and length of the CNT. Given the power law, this could take considerably longer than most experiments would last. Lucas et al. propose a power law exponent *m* = 0.21 for 500 nm long CNT that were shortened to about 200 nm length by 30 min sonication with 10–160 W. The argument for why this power law was found would be contradictory to [62], but the possibility that buckling could be the reason for the power law exponent of 0.2 is also granted. A variation in the power law display is suggested that allows one to better estimate the length reduction by plotting in a double logarithmic scale the length *L* as a function of sonication energy, *E = t × P*, with *P* the sonication power, and thus *L(t)~E^−m^*. This relation allows one to scale measurements with different input power and find the common underlaying power law exponent more easily. It also shows the continuous fragmentation of shorter CNT at a lower rate much better [37]. As an example, the MWCNT length as a function of time and energy is depicted in Figure 4.

A power law dependence on the sonication energy was also reported by Chapkin et al. SWCNT with about 1.4 nm diameter and 1000–5000 nm length were sonicated by a horn sonicator with increasing sonication energy. The length of the CNT was reduced and the length distribution narrowed down. A power law exponent of *m* = 0.41 was found [64], which according to [62], would hint at rupture rather than buckling.

A lower limit for the CNT length by scission due to rupture was suggested by Huang et al. They also assume drag forces acting as tensile stress on the nano-fibers that are oriented in a radial direction towards the cavitation bubbles. Sonication of MWCNT (CVD produced, 60–100 nm diameter, 5–15 μm length), protein filaments, and silver nano-wires (300 nm diameter, 10–25 μm length) was performed for 7 h, with about 190 W and an average power density of >60 W cm^−2^. It was shown that the limiting length depends mainly on the diameter and the tensile strength of the fibrils. A lower length limit of about 2–6 μm was found, for example, for the MWCNT. It is also stated that the length distribution narrows down considerably with sonication time. This method is actually used to measure the diameter and length after sufficiently long sonication time and calculate the tensile strength of the nano-particles therefrom. It is stated that the driving mechanism for shortening will be tensile rupture in cases where the dimensions of the sonication bubble would be of a similar scale or bigger than the typical length of a filament in question [65].

For the special type of more weakly bound bamboo-shaped MWCNT, Jang et al. report on an exponential decay of CNT length with sonication energy, that is, time of sonication with a 150 W (28 kHz) sonication bath. It is reasoned that the weak axial bonding in such structures might lead to different scission mechanisms, other than buckling or tensile failure, mainly at the middle of the CNT. In this study, a minimum scission length is also observed [66].

#### 2.2.2. Methods for Monitoring Damage

Several methods have been applied to monitor the effect of chemical change or damage to CNT. This section shall give a short overview of this experimental tool-box.

**Figure 5:**
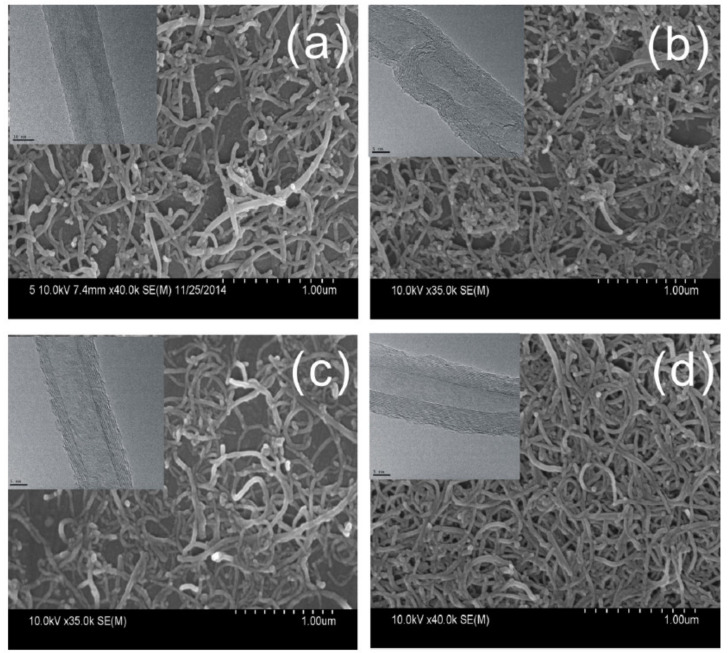
Example of TEM and SEM micrographs; (**a**–**d**) indicate the effect of different functionalization procedures (acid treatment with ultrasonication) to MWCNT, cited from [15].

Transmission electron microscopy (TEM) is mainly used to study structural integrity of the walls of MWCNT and to directly see damage or fragmentation of CNT in general [15,40,60,61,66]. The proper selection of magnification also allows one to monitor diameter distribution and even length distributions [67]. Scanning electron microscopy (SEM) is often applied in order to study agglomerate size after dispersion [27,58], which is also regularly performed by light microscopy (LM) [61], or to examine CNT location in a matrix (e.g., [39,41,57]). Even a single MWCNT could be resolved, probably by higher visibility due to surface charge [35], or by having a diameter of about 100 nm [65,66]. An example of TEM and SEM characterization of MWCNT affected by different functionalization procedures including sonication is depicted in Figure 5.

**Figure 6:**
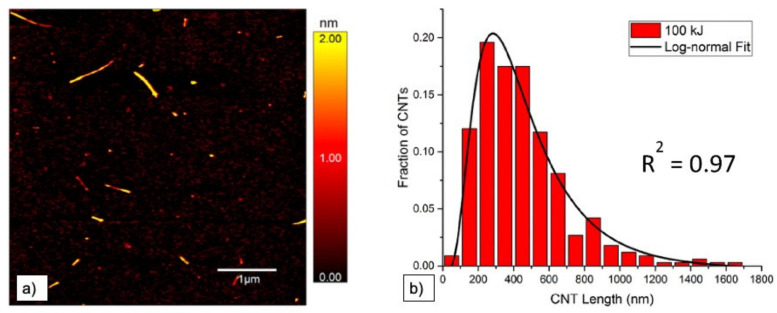
(**a**) Example of AFM image with visible height profile and observable length of SWCNT after sonication with a tip sonicator at 100 kJ and (**b**) the corresponding length distribution, from [64].

Atomic force microscopy (AFM) is used to study diameter and length of CNT. It is often not only used to measure mean diameter and length values, but to determine length and diameter distributions, which give more precise information on the effect of dispersion [25,36,64,68,69]. All three microscopy techniques (i.e., TEM, SEM, and AFM) can in general be used to determine length and diameter of nano-particles on different length scales. Measurements can directly be performed and pictures evaluated automatically. Usually, results are displayed in histograms displaying the size distribution (e.g., [38,48,64,67]). An example of an AFM image together with the corresponding length distribution is depicted in Figure 6, cited from [64]. A second example of such a length distribution determined by AFM, taken from Hennrich et al. [36], can be found further below.

Raman spectroscopy is used to determine surface damage of the nanotubes. The characteristic D-band at 1340 cm^−1^ is a defect-induced feature attributed to dislocation defects, representing the carbon atoms on the surface showing sp ^3^ hybridization. The characteristic G-band at 1580 cm^−1^ related to in-plane vibrations of the graphene layers in graphite or nanotubes thus refers to the graphitic structure of the surface. The G-band is also reported in more detail to split into G^+^ (1589 cm^−1^) and G^−^ (1568 cm^−1^), detailing the in-plane vibrations along the nanotube axis and around the circumference, respectively. Another band reported in the literature is the D’-band (1620 cm^−1^), which optimally has to be separated from the G-band.

**Figure 7:**
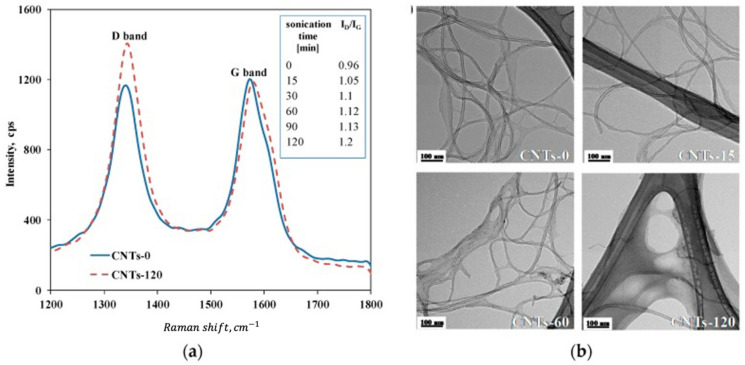
Effect of ultrasonication on surface changes of CNT, cited from [70]: (**a**) shift of D-band to G-band during ultrasonication process, representing the concentration of defect site content on the CNT structures; (**b**) TEM images showing alterations of ultrasonic-treated CNT compared to untreated ones.

The G-band is independent of the presence of defects. Hence, for a qualitative analysis of defects, the ratio of the D- and G-band (G^+^-band) intensities I_D_ and I_G_ is calculated, (i.e., I_D_/I_G_), which is thus a measure for the concentration of defect site content on the CNT structures, or the relative disorder of MWCNT structures [15,70,71]. An example of the typical G- and D-band changes due to sonication is depicted in Figure 7. Sometimes, the fraction of G-band intensity I_G_/(I_D_+I_G_) is also determined [66]. One has to consider, though, that the D-band might also be influenced by the amount of amorphous graphite in a sample [25]. These carbonaceous impurities might only allow it to follow a relative change and even be changed as well due to sonication treatment.

**Figure 8:**
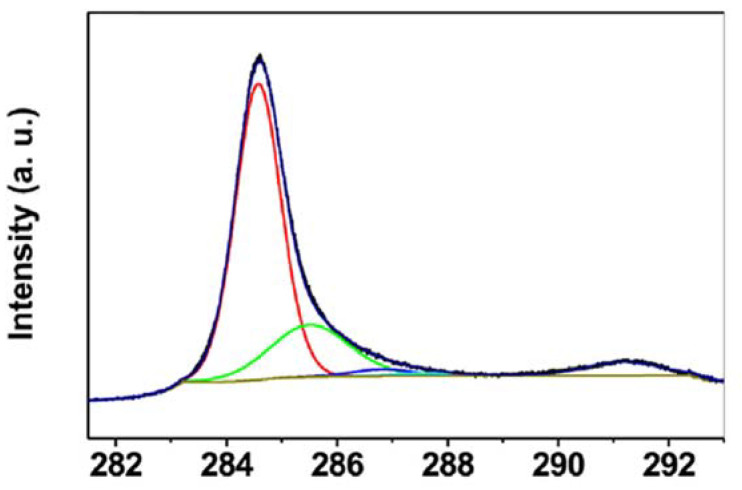
Example of the XPS C1s peak of MWCNT, showing the C=C peak sp^2^ (red), the C-C peak sp^3^ (green), a C-O peak (blue), and the π–π* transition (situated at 291 eV, but not indicated), cited from [73].

X-ray photoelectron spectroscopy (XPS) is used to determine the proportion of non-carbon atoms, like nitrogen or oxygen, within the CNT, and in general, the chemical binding states. It is therefore mainly used to follow oxidation, functionalization, or doping of CNT [15,17,67,72]. Surprisingly, until now it has not been applied to study the sonochemical effects of ultrasound treatment to CNT directly. The C1s peak can be deconvoluted in up to six sub peaks, relating to, for example, C-C, C=C, C-O, and C=O bindings. The binding energy of 284.5 eV was reported to correspond to sp^2^-hybridization in graphite-like carbon, and the peak at 285.5 eV was reported to correspond to sp^3^-hybridization and is interpreted in relation to defects [15,73]. An example of the deconvolution of the C1s peak with contributions of C-C, C-O, and π–π* is depicted in Figure 8.

Two methods seldomly used in relation to damage during ultrasonic dispersion are X-ray diffraction (XRD) and Fourier-transform infrared spectroscopy (FTIR). XRD can be used to investigate the integrity of the graphitic nature of, for example, MWCNT [72,74]. FTIR is used to investigate changes in bonding nature in the nanotubes, like -C=C or possibly induced -OH [15,27,75].

#### 2.2.3. Methods for Monitoring the Dispersion State

**Figure 9:**
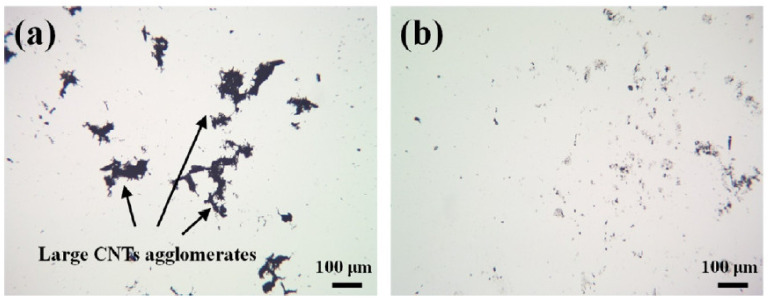
Example of light microscopy for monitoring the agglomerate size: (**a**) large agglomerates of MWCNT after sonication with 25 J/mL; (**b**) small agglomerates after sonication with 400 J/mL [41].

Several methods are used to determine the dispersion state achieved by sonication, that is, the amount of single CNT rather than damage to the CNT. These are described as follows. Besides the aforementioned microscopical methods AFM and SEM, light microscopy (LM) is also suited to determining the size of CNT agglomerates, and thus monitoring the success of breaking up agglomerates and separating the CNT into the solvent system [20,41,61]. For the evaluation, automated image evaluation programs can be applied, resulting in size distributions and even indicating if agglomerates are more elongated or round [24]. An example of LM micrographs is depicted in Figure 9.

**Figure 10:**
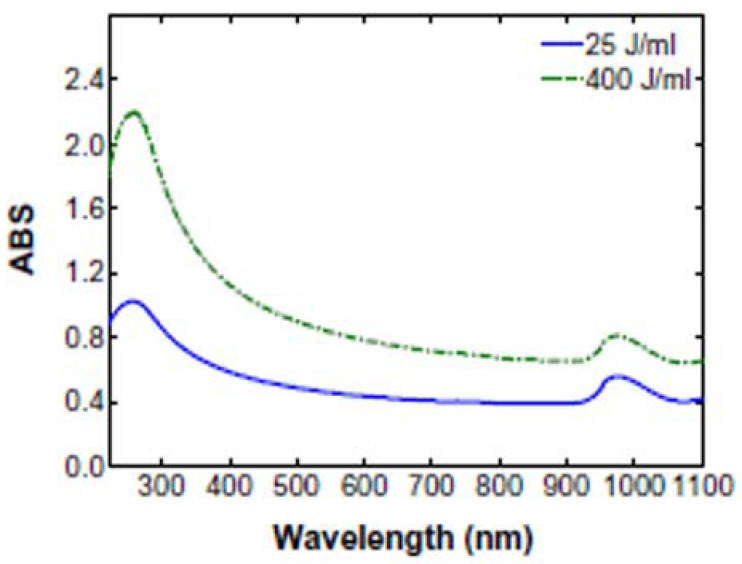
Typical UV–Vis spectrum of MWCNT with the two absorption bands at 253 nm and 970 nm [35].

Ultraviolet visible light absorption spectroscopy (UV–Vis, also UV–vis-NIR) is used to detect the amount of well-dispersed CNT, since the absorption of single CNT is much more pronounced in the range from 200–1200 nm, which is not the case for agglomerates and bundles. SWCNT show several absorption bands at about 550 nm, 972 nm, and 1710 nm; MWCNT, at 253 nm and about 970 nm, respectively [35,36,76,77]. An example of the two MWCNT-related peaks is depicted in Figure 10. Bundling of nano-tubes can also result in broadening of the peaks and a red shift in the position. This can be used for estimating mean diameters [25,48].

Dynamic light scattering (DLS) is usually applied to study the size of CNT agglomerates, and is thus very well suited to follow the success of the dispersion process. In the literature, different names can also be found, such as depolarized dynamic light scattering (DDLS) or liquid mode laser diffractometry (LMLD), the latter being a special application with DLS in flowing liquid. Low amount of CNT is a prerequisite in order to allow for meaningful signals [36]. While the length of individual CNT is only accessible indirectly, and therefore damage to single CNT is not accessible, the effect of sonication to the agglomerates and CNT bundles can be studied in detail. DLS can give valuable integral information on size distributions [56,78].

**Figure 11:**
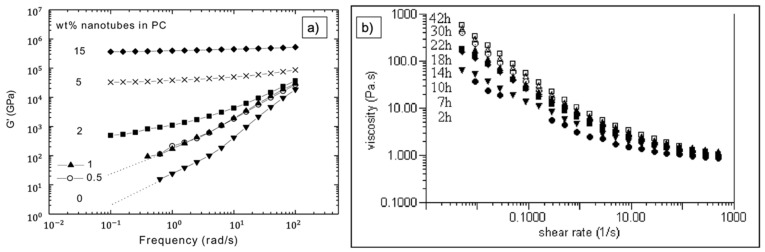
Example of results from rheology experiments. (**a**) Depiction of a frequency sweep test showing the frequency dependency of the storage modulus G’ for various CNT filler grades. A frequency independence at low frequencies is related to the formation of internal networks [79]. (**b**) Rotational test evaluating the shear thinning behavior with increasing sonication time. A higher viscosity and more pronounced shear thinning behavior is referred to as MWCNT separation. Reprinted with permission from [83], Copyright (2007) The Society of Rheology.

Rheology is applied to gain information on the viscosity and network properties of a liquid or polymer. The values and the changes of storage modulus G’ and loss modulus G’’ are studied and relate to a more viscoelastic or more liquid behavior, respectively. These parameters change due to well-dispersed CNT, in contrast to the same filler grade of CNT present in agglomerates (Figure 11a). Standard tests are amplitude sweep, frequency sweep, and rotational test for, for example, thixotropy. Dispersed CNT tend to form a percolation network, especially for higher filler grades, which results in an increase in viscosity (Figure 11b) and of the storage modulus G’. Another parameter that is often considered as well is the damping factor tan (δ) = G’’/G’. It is reported to be smaller than 1 for good dispersions. In general, shear thinning behavior is reported [24,61,79,80,81,82].

Two methods seldomly used to determine the state of CNT dispersion are Raman spectroscopy and conductivity measurements. Raman spectroscopy is generally used in order to determine damage to the CNT surface (e.g., [70]), but is also reported for monitoring the amount of single CNT by the ratio of G^−^/G^+^ [84]. Conductivity measurement is also scarcely applied. A conductivity probe can be either used directly during sonication or separately. The conductivity is low for big non-entangled agglomerates and increases with increasing amount of well-dispersed nanotubes, which start forming a network of dispersed fibrils. Thus, this method can be used to follow the degree of dispersion and decide when a state close to optimal CNT dispersion is achieved [61,85].

Two techniques that proved useful in course of the reported experiments shall be notified: centrifugation to remove, for example, larger agglomerates, and size exclusion chromatography (SEC) for length selection [36].

Table 1 summarizes the described methods for determining the dispersion state of CNT and possible damage (scission, change in the surface structure or generation of side groups, for example, due to functionalization) and gives the relevant citations for further reading.

### 2.3. Effects of Ultrasonication Documented in Literature

#### 2.3.1. Reported Shortening of Nano-Fillers

It is highly important to know what effect a specific set of ultrasonic dispersion parameters, like power, pulse—pause operation, solvent system, sonication time, and so on, has with respect to a given species of nano-particle. Therefore, several publications that report on the scission due to sonication are described in more detail in the following.

Sesis et al. investigated the influence of stable and inertial cavitation through experiments in a sono-reactor. Stable cavitation leads to a chemical modification of the surface of the CNT, while inertial cavitation causes better exfoliation, but also pronounced length reduction. They tested the shortening and surface damage of the nanotubes at different power inputs (100 and 200 W) and different critical micelle concentrations (cmc) (30% and 300%). Length reduction effects were analyzed by atomic force microscopy and found to be highest for 200 W and 300% cmc, with an average of 36% length reduction [38].

**Figure 12:**
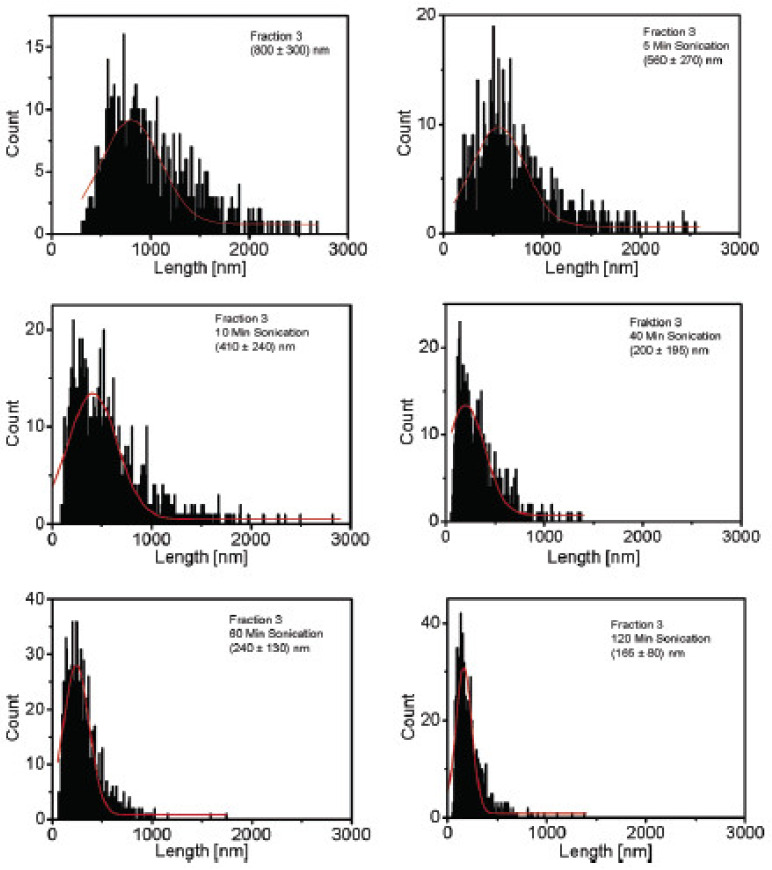
Shortening of SWCNT by sonication. The shift of the length distribution to smaller values and the narrowing down of the length distribution is visible. Reprinted with permission from [36], Copyright (2007) American Chemical Society.

Hennrich et al. applied sonication with a tip sonicator with an output power of 20 W at 20 kHz for up to 2 h in a cooling bath. The length of sonicated SWCNT was determined quantitatively by AFM and qualitatively by photoluminescence. The resulting CNT length distribution changes with sonication time are depicted in Figure 12. Shortening was observed already after 5 min sonication time, and length *L* was reported to decrease with a power law dependence on the time *t: L~t*^−0.5^. Additionally, a lower length limit to this scission process was found, depending on the tube diameter, length, and liquid parameters. CNT would not get shorter even at much longer sonication times after this limit was reached. It is indicated that the shortening process starts immediately after the sonication starts [36].

Wu et al. used carboxylated multi-walled carbon nanotubes and dispersed them in Milli-Q water by a sonicator at 100 W and 20 kHz for 7 h under a 25 °C water bath. Length reduction was investigated by SEM analysis. The length of the MWCNT was reduced due to the functionalization process, and further because of the ultrasonic dispersion, from a mean length of 1167 nm to 902 nm, and finally to 158 nm, respectively [87].

Ruan and Jacobi dispersed MWCNT in ethylene glycol including gum arabic as a dispersant with a tip ultrasonicator at 150 W and 20 kHz in continuous mode and in pulse mode (0.8:3.2 s pulse/pause). They compared the impact of these two modes on the thermal conductivity of otherwise identical samples. Five readings were averaged with a two-σ precision limit of ± 3%. It was found that the sonication mode had almost no impact on the thermal conductivity within the chosen limit, and thus the pulse mode sonication was chosen as standard procedure. The thermal conductivity increased with increasing sonication time and energy input. LM and TEM revealed shortening of the nanotubes and a reduction in the aspect ratio with increasing sonication time and sonication energy [88].

Dassios et al. dispersed SWCNT with the help of solvent using an ultrasonicator with sonotrode with a power ranging from 100 W to 400 W. They analyzed CNT agglomerate size reduction by liquid mode laser diffractometry, which also allows one to measure the agglomerate size distribution. It was found that the energy transferred to the CNT in suspension not only depends on the power setting, but also on the immersion depth of the sonotrode. The sonication energy transfer at a lower rate of 5.4 kJ/min was not sufficient to reduce the agglomerate size significantly below 11 μm within 30 min (162 kJ of applied energy). Agglomerate dimensions measured below 11.2 μm are interpreted as a signal of either actually small agglomerates or possibly well-dispersed CNT. Sonication with 7.7 kJ/min for 90 min (231 kJ of applied energy) results in a main size distribution around 1–2 μm. Sonication for 120 min did not reduce the agglomerate size or narrow down the size distribution any more [56].

Arrigo et al. analyzed the shortening of CNT in an ultrasonic bath (power 260 W) through evaluation of the distribution of the hydrodynamic diameter *D_h_* evaluated by dynamic light scattering at different sonication times. Additionally, rheological tests and micro-Raman spectroscopy were used to detect possible defects. With increasing sonication time, *D_h_* decreased linearly from 150 to 30 nm. The corresponding length of CNT was calculated to decrease from about 400 to 50 nm. Rheologically, it was concluded that the shortening of the nanotubes also counteracted the formation of a percolation network [70].

Yu et al. worked on optimizing the dispersion process for single-wall carbon nanotubes (SWCNT) in an aqueous solution of 1 w/v% sodium deoxychlorate (DOC) by varying the ultrasonication power from 20 to 120 W at 20 kHz in combination with an immersion depth of 25 mm (sample volume 50 mL). The sonication time ranged from 10 to 120 min in intervals of 10 min. UV–vis-NIR was used to analyze the dispersion quality. The diameter and length of the SWCNT were evaluated by AFM. They found that both power and sonication time have an influence on the shortening, but a sufficient power is needed to reach a well-dispersed state. Increasing the sonication time in a well-dispersed solution leads to a further reduction in length and diameter [69].

Chen et al. investigated the influence of applied ultrasonic energy on the shortening effect and the reinforcing efficiency of CNT. They dispersed the CNT in distilled water and a polyacrylate-based dispersing agent at an output power of 150 W at 20 kHz. Samples were analyzed after different energy inputs (25 J/mL, 75 J/mL, 150 J/mL, 250 J/mL, 400 J/mL) by SEM and UV–vis spectroscopy. The shortening effect was evaluated by measuring the length of the tubes determined in the SEM images and fitting the data with log-normal and Weibull distributions. They found that the histograms of the measured tube lengths appeared to be bell-shaped under log scale, and the broadness of the distribution decreases with increasing energy input (standard deviation from 605 nm to 273 nm). Further, the peak of the distributions shifts from 265 nm (75 J/mL) to 216 nm (400 J/mL) with increasing energy. At lower energy input (under 75 J/mL), the volume distribution remained almost the same. This was interpreted to mean that below this energy level, the ultrasonication process mainly contributes to exfoliation and does not lead to considerable shortening of the tubes [35].

Huang et al. refer to previous studies [36,89,90] that reported on sonication-induced length reduction, where an initial broad length distribution of the CNT changed to a narrower length distribution by the sonication process. After, the shortening effect the CNT showed a constant modal length throughout the prolonged sonication exposure. Huang et al. interpreted this as a length reduction through mechanical shearing from the fluid flow, rather than by thermal or chemical effects. Further, they developed a simple model describing the shortening of nano-fillers during hydrodynamic stresses caused by sonication-induced cavitation. For MWCNT, they found a length reduction to 2–6 µm after 3 h sonication (pulsed-pause setup 5 sec on/3 sec off, power density >60 W/cm^2^), and after additional 7 h sonication, the reduction was only minimally affected. The proposed model predicted a length of 3–5 µm, which was in good agreement with the measurements, suggesting that the model is a versatile tool to determine the tensile strength of nanofilaments by length measurements. This also indicates a lower limit to length reduction, dependent on the properties of the filaments [65].

Badaire et al. used depolarized dynamic light scattering (DDLS) to analyze the ultrasonic-induced length reduction of CNT bundles through experiments at different power (20 W and 40 W) and different sonication times. Increasing power led to a quicker length and diameter decrease and narrower size distribution. After 120 min at 20 W, SWCNT bundles showed a mean length of 1146 ± 187 nm and a mean diameter of 12.1 ± 2.5 nm, while after 120 min at 40 W, the length was reduced to 822 ± 83 nm and the diameter to 2.9 ± 0.7 nm. They also performed conductivity measurements. The resistivity of the solution increased with increasing sonication time and input power: 5 min sonication at 20 W with 20 min high shear mixing resulted in a resistivity of 0.4 Ω cm, 15 min sonication at 20 W with 20 min high shear mixing already showed a resistivity of 14 Ω cm, and sonication for 120 min at 40 W followed by 20 min high shear mixing resulted in 32.1 Ω cm [78].

Tian et al. presented a two-way approach to functionalize MWCNT with assisted ultrasonication. MWCNT were ultrasonicated for 40 min with NaCO_3_ and H_2_SO_4_. In the second step, KMnO_4_ was added, and the solution was ultrasonicated for additional 2 h. For comparison, the KMnO4 was also added without sonication. They found that the pristine CNT had a broader size distribution centered at 50 µm, while the ultrasonically treated and modified MWCNT showed a narrower size distribution, with a center at 10–15 µm [15].

Fuge et al. investigated the shortening of N-doped MWCNT and concluded that these are more prone to shortening by sonication. It was stated that this effect is even enhanced for higher amplitudes, while longer sonication times do not increase the rate of shortening for a given ultrasonication amplitude [67].

An overview of the described effects of length reduction together with relevant sonication parameters can be found in Table 2.

#### 2.3.2. Reported Defects and Changes in Surface Chemistry

Arrigo et al. analyzed surface changes by Raman spectroscopy at different sonication times. The ratio I_D_/I_G_ increased with increasing sonication time from 0.96 for the untreated samples to 1.2 after 120 min of sonication, indicating a progressive damage mechanism inducing structural defects on the surface over time [70].

Jung et al. pre-treated MWCNT using supercritical CO_2_ and ethane fluids prior to dispersing them in an SDS solution via ultrasonication. The dispersion process was accomplished in a sonication bath at 60 W and 28 kHz for up to 200 min. For both pristine and scEthane-treated CNT, the band-ratio I_D_/I_G_ gradually increases during the sonication process, indicating more damages with increasing sonication time. Additionally, the pristine CNT showed a higher damage rate compared to the supercritical-fluid-treated samples, namely, the I_D_/I_G_ ratio increased from approximately 1.37 to 1.67 for pristine CNT and from approximately 1.35 to 1.54 for treated CNT after 50 min of sonication [91].

Cheng et al. analyzed their samples via Raman spectroscopy after sonication with a tip sonicator with 195 W output power in different solvents for up to 220 s. They found increasing values of the I_D_/I_G_+ ratio with increasing sonication time. Results differed for different solvent systems (I_D_/I_G_+ ratio increases by a factor of 1.4 or 1.7 depending on solvent system). This was related to an induced tube length reduction, rather than surface damage. They argue that in previous studies, an increase of the band ratio is also connected to nanotube scission. They are the only known publication to relate Raman spectroscopy results with length reduction; therefore, they are reported in context with the other Raman-related publications in this section, rather than together with the publications dealing with length reduction. In the same publication, they investigated if the ratio increase fits to the square root of the sonication time *t*, since the sonication-induced length reduction follows in some cases the decay of *t*^−1/2^ (according to Hennrich et al. [36]). Measurement points of I_D_/I_G_+ were taken in a timeframe of 20–220 s ultrasonication time. The largest increase in the ratio I_D_/I_G_+ was found for both solutions in the first 60–80 s; afterwards, the ratio I_D_/I_G_+ approaches a plateau [92].

Tian et al. studied the formation of functional groups by acid treatment and simultaneous sonication. With Raman, they found increasing values for the relative intensity ratio of I_D_/I_G_ from 0.84 to 1.14 for oxidation/ultrasonication for 40 min. Unfortunately, the sonication power and frequency were not given, but the publication is anyhow outstanding, since acid treatment was deliberately applied in order to functionalize the nanotubes. They also applied FTIR and XPS to confirm oxygen-containing functional groups induced by sonication [15].

Lu et al. suspended CNT in CH_2_Cl_2_ and applied sonication at comparable low power of 17 W for 5 to 20 min. Even at these low powers, an increased I_D_/I_G_ ratio was observed. They further evaluated via electron spin resonance (ESR) spectroscopy a g value of 2.000 for untreated CNT, which is assigned to carrier electrons in the conduction band of the graphite layers and is broadened due to sonication. The authors related this to defects that disturb the regular structure of the layers [71]. In contrast to this, Koh et al. report that sonication of SWCNT in a non-acidic environment for up to 6 h with a power of up to 80 W in a sonication bath did not show any change in the D- and G-band ratio, and concluded that the covalent surface of the CNT might not have been changed considerably [93].

Raman spectroscopy turned out to be a powerful method to detect surface damages and changes through evaluating the D–and G-band peaks, which represent the carbon atoms on the surface showing a sp^3^ hybridization and those that are related to the sp^2^ hybridized carbon atoms, respectively. An overview of the citations dealing with surface changes and induced surface damages of CNT due to sonication investigated by Raman can be found in Table 3, together with the applied sonication parameters.

Two other methods for damage characterization besides Raman spectroscopy shall also be mentioned. FTIR analysis was used to characterize the content and insertion of functional groups during the sonication process. Tian et al. detected and analyzed four different characteristic peaks at 3424, 1727, 1631, and 1102 cm^−1^. Those peaks were assigned to -OH, -COO, -C=C, and -C-OH stretching vibrations, respectively. They found for neat MWCNT no identifiable functional groups, while the ultrasonicated samples showed oxygen-containing functional groups [15]. Mukhopadhyay et al. reported on damages to regular graphene sheets after 4 h of sonication (power input 2 W) detected via HREM and a decrease of intensities in the solid-state UV–VIS and FTIR spectra with increasing sonication time. After 24 h, almost all peaks vanished, which was interpreted as a complete conversion of graphitic layers into amorphous carbon [94].

#### 2.3.3. Monitoring the Dispersion State of CNT

**Figure 13:**
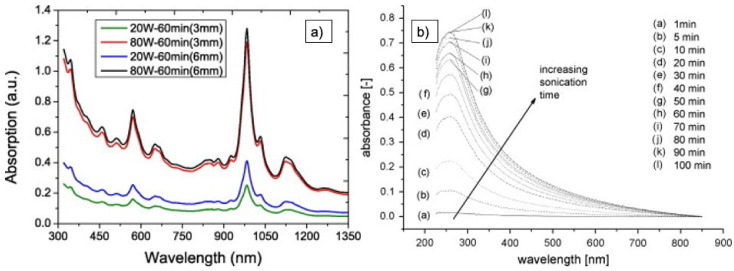
Examples of the application of UV–vis absorption spectroscopy. (**a**) Spectra from SWCNT for two different amplitudes and two different sonication tips, respectively [69]. (**b**) Spectra for functionalized MWCNT dispersed in deionized water + surfactant for increasing sonication time [70].

Yu et al. found that the magnitude of the two significant peaks at 551 nm and 976 nm in UV–vis-NIR spectra of (6,5)-SWCNT is dependent on the sonication power and time: application of a sonication power of 80 W or higher for, for example, 60 min resulted in a massive increase of both peaks. They correlate these spectral changes to an increase of individual SWCNTs and small-diameter SWCNT bundles in solution—an effect that is enhanced by application of a centrifugation process after ultrasonication. They also showed that a sonication tip with a bigger diameter gives rise to a higher absorption spectrum (see Figure 13a), which is related to a higher amount of dispersed CNT for the same sonicator amplitude and sonication time [69].

Rausch et al. investigated the interaction of different CNT surface chemistry with the solvent system during sonication. They used different batches of MWCNT from Nanocyl together with deionized water and different surfactants, which were sonicated with a 140 W tip sonicator for different time intervals. XPS and FTIR were used to characterize the surface chemistry of the CNT before sonication, and UV–vis was used to follow the dispersion. An increase of the absorbance by about 90% was found when the sonication time of CNT was increased from 5 min to 100 min (see Figure 13b). They also conclude that for each surface chemistry, the optimal surfactant has to be found, since absorbance, and thus dispersibility, is influenced by the chemical interaction to some extent [75].

Sesis et al. analyzed UV–vis-NIR spectra and calculated the area ratio of the E_22_ resonance peak at 570 nm. They found that with increasing power and sonication time, the absorption resonance ratio increased, indicating that the amount of dispersed CNT was increasing. Further, a higher surfactant concentration (300% cmc) resulted in a higher exfoliation of CNT. The dispersion efficiency in this case was 25% for 100 W and 80% for 200 W power input [38].

Tian et al. report that the increase in the resonance ratio (area fraction of resonant band at ~567 nm to all bands from 436–709 nm) can be used to follow the exfoliation of SWCNT. They report that exfoliation of the SWCNT, takes place in the first hour of sonication. A horn sonicator (Dismembrator Fisher Scientific model 500) with 35% power output was used. Furthermore, very long sonication times of 3 days lead to an increase in background signal with a constant resonance peak intensity, meaning a decrease in the resonance area. They interpreted this as a possible increase of damages induced to the nanotubes, under the assumption that there are enough CNT available for dispersion. In the next step, they tested various surfactants and tried to maximize the resonance ratio for each type by varying the dispersion parameters [86].

Chen et al. applied UV–vis to follow the dispersion state of MWCNT and stated, based on the Beer–Lambert law, that the concentration of dispersed CNT is proportional to their absorbance. Thus, the measured absorbance was converted to a relative concentration of dispersed CNT. The absorbance and thus the concentration of dispersed CNT was found to increase with the applied ultrasonic energy. From 25 J/mL to 400 J/mL, the absorbance increased from 0.4 to 0.8, while the normalized CNT concentration increased from 0.5 to 1.0. They interpreted the change in the signal as exfoliation rather than fiber breakage. The normalized CNT concentration increased from 0.5 to 0.9 between 25 and 150 J/mL, and only increased another 10% between 150 J/mL and 400 J/mL. After exceeding a certain energy level, the absorbance remained at an almost constant value [35].

Ganapathi et al. analyzed UV–vis spectra, which showed increasing absorbance values for up to 30 min sonication. This was related to a debundling of CNT. A further increase of sonication time to 60 min or more, and thus increasing sonication energy input, resulted in a decrease of absorbance anyways, indicating a partial re-agglomeration or bundling of the MWCNT. After 15 min sonication, the absorbance value at 500 nm was between 0.45 and 0.65, and after 30 min it increased to 0.75–0.85, but after 60 min it decreased again to 0.58–0.82, and after 90 min it became 0.55–0.9. Furthermore, Ganapathi et al. interpreted a narrower distribution of absorbance values as an indication for better CNT distribution within the dispersion [61].

Rheological tests deliver insights into the long-term stability of dispersions. Pötschke et al. found that G’ and G’’ are nearly independent in the low-frequency range for MWCNT. The slopes of these curves decrease with increasing nanotube content. These findings are connected to the formation of internal structures, which is only possible if the CNT are exfoliated. This publication serves as a great overview of characterizing dispersion states via rheological tests, despite the fact that these dispersions were not produced via ultrasonication [79].

Fan et al. tested different types of dispersions, using MWCNT mixed in acetone and epoxy and including an evaporation process afterwards, either using a magnetic stirrer or an ultrasonic bath. They found that the shear viscosity can be related to the MWCNT separation: while the storage modulus is more sensitive to CNT interconnections, the shear viscosity is more sensitive to CNT separation. A higher flow resistance is related to separation of CNT into the matrix, and lower values represent MWCNT that are found not in aggregates. The shear viscosities were found to be highest in the ultrasonic suspension. With increasing ultrasonication time, these values also increased. This indicates that differences in the separation and interconnection of MWCNT could be characterized by rheological properties [83].

Kim et al. dispersed neat and various functionalized (amine-treated, acid-treated, plasma-treated) MWCNT in ethanol for two hours, and afterwards in heated epoxy for one hour via ultrasonication, followed by an evaporating process for five days. They observed shear thinning behavior for all samples. This behavior was more pronounced for the functionalized samples. The shear viscosity was highest in the various functionalized samples (amongst them, the plasma-treated ones showed the highest values) compared to neat MWCNT. Storage and loss modulus also showed the highest values for plasma-treated MWCNT compared to the untreated ones, and the increase rate of those values with frequency was lower for modified ones. This is related to a better interfacial bonding and the formation of a network-like structure between the resin and the CNT, leading to a better separation of the nanofiller [80].

Ganapathi et al. characterized the viscoelastic properties as a function of frequency with a testing range of 0.1 to 100 rad/s with a strain amplitude of 1% at 80°C. They found that from 15 to 30 min sonication, the storage modulus increases over time and frequency, indicating that an effective particle-to-particle network was formed. Nevertheless, with increasing sonication time (60–90 min), the network and the distribution of the CNT was negatively affected, as was also documented by UV–vis (see above [61]).

Dassios et al. used LMLD to characterize the dispersion state. They analyzed MWCNT suspensions ultrasonicated with different energy inputs: 5.4, 27, and 162 kJ (related to 5400 J/min applied for 1, 5, and 30 min sonication time). The suspensions were compared directly after dispersion and after a period of three days’ rest to allow for thermodynamic equilibrium. The peaks of the size distributions and the calculated polydispersity index showed an energy input insufficient for a complete disentanglement of the nanotubes: peaks size below 12 µm were related to entangled states and were unaffected by ultrasonication. Above this threshold, the size distribution peak appears at different locations for different energy inputs. It was further found that a higher energy input rate of 7700 J/min for 90 min was sufficient to disperse MWCNT homogeneously in an aqueous suspension [56].

An overview of the described methods for investigating the dispersion state of CNT can be found in Table 4, presenting information on the solvent system, the type of CNT, the sonication power, sonication time or energy applied, characterization method, dispersion result, and respective citation.

## 3. Discussion

### 3.1. General Remarks

A lot of publications report on the effects that ultrasound amplitude, frequency, and sonication time have on the dispersion quality, and subsequently on the products’ properties (e.g., composites), but do not consider damage effects. The effect that shear and sonochemistry might have on the nano-particles is often not considered. This could have an unknown influence on the experimental outcome. Although these influences might be small, considering such effects could help to better understand the complex nature of nanotube application. Thus, it would always be beneficial to apply some of the afore mentioned methods for damage monitoring to clarify:

- If and what damage is done to the nano particles (i.e., surface change or scission) and to what extent this happened.
- In this context, it might be considered if a better dispersion/break-up of agglomerates is perhaps partly caused by breaking the entangled fibers. These shorter and likely disentangled fiber fragments could easily be moved from the agglomerate, thus contributing to a better dispersion.
- Subsequently, it will be important to investigate if a change in material properties of a final product (e.g., a composite) is caused solely by better dispersion (i.e., smaller agglomerates and more well-dispersed nano-fibers) or if a shortening of a considerable number of nano-fibers has an additional effect on the results.

Any combination of methods that allows one to determine the dispersion state and a possible length reduction, and optimally also surface damage, would be applicable. For example, a combination of LM, SEM, and FITR, or DLS, TEM, and XPS. Many of the presented research papers gave a very concise and valuable insight into the application of such methods and the different possibilities of combining them. Only a few of them deal with a multitude of methods. Examples of such a combination of several methods, to characterize CNT dispersion and integrity together with methods for further product characterization, can be found in the following publications: Cuong et al., applying Raman, XRD, XPS, TEM, FTIR, nuclear magnetic resonance (NMR), thermo gravimetric analysis (TGA), and differential thermogravimetry (DTG) [72]; Blanch et al., applying UV–vis-NIR, Raman, AFM, TEM, and elemental dispersive X-ray spectroscopy (EDX) [25]; and Tian et al., applying FTIR, XPS, Raman, TEM, SEM, and DLS [15]. Although two of these apply, for example, FTIR, interestingly, FTIR is not often used for measurements of changes in CNT due to sonication [15,27], but rather in relation to epoxy resin, to follow curing or degradation reactions [70,72]. The reason might be the effort of preparation of dry pellets for this measurement method.

To monitor surface changes, Raman spectroscopy is mainly applied. From the presented publications, Tian et al. report on considerable damage of the CNT surface, measured by Raman spectroscopy even at low powers of about 17 W, while Koh et al. suggest that at powers up to 70 W, no damage could be observed by Raman spectroscopy [15,93]. There are several experimental differences that could lead to this discrepancy. Most importantly, the solvent system is different. Tian et al. aim at functionalization by application of an acidic environment. Another difference is the application of a tip sonicator in the first case, while the 70 W in the latter case was applied by a sonication bath. Since the power density in a sonication bath is lower than with a tip sonicator, this could again be a hint that reducing the power density by plate sonicators or even using a sonication bath might take a longer time for dispersion of nanotubes, but might keep nanotubes more intact (i.e., avoid length reduction and surface damage).

Findings of Sesis et al. [38] also indicate that lower power densities, as achieved by reduced input power and broader transducer tip, would result in surface damage, while higher power densities result in rather fractured CNT. Lower power densities favor stable cavitation, while higher power densities favor transient cavitation. A similar finding was reported by Islam et al.: longer sonication times with lower power left CNT rather intact, whereas shorter sonication time with a higher power of tip sonicator resulted in clearly shorter nanotubes [68]. In the model of [62], no ultrasonic power was introduced, while the measurements of [36] include sonication power. The power put into the system will affect the scission mode in two ways. One is that the cavitation type changes from stable to transient, and with this, the bubble size and lifetime, and therefore the force of the shock wave acting on the surrounding fibers, changes. Then, the maximum amplitude of the shock wave changes as well, and with this, the maximum force acting on the nano-particles changes. This again changes the scission mode for a given nano-particle system [36,37,38,62]. Other influences in this complex system are the physical properties of the nanotubes or nano-fibers themselves and the type of solvent system. The fiber diameter directly influences the CNT stiffness and thus the point at which the scission mode changes from buckling to rupture, evident in the power law dependence of the CNT length decreasing with time [62]. The solvent itself has a great influence on the cavitation and flow behavior around the nano-particles, and therefore on the scission mode [38,75]. Moreover, the immersion depth of the sonicator into the liquid influences the effect on the nano-particles [56]. Low power sonication that results in stable cavitation more likely features standing waves in the sonication containment, which could result in re-agglomeration, if not taken into account [24].

### 3.2. Perspectives

Considering all this, it is not straightforward to conclude on the scission mode and the shortening of CNT during dispersion from one factor, like input power or sonication time alone; all these factors should be taken into account. This will hardly be possible during standard sonication. There are two ways to deal with this. Preliminary tests can estimate damage to the nano-fillers, dependent on the sonication parameters. The results would allow the selection of those parameters that cause the least damage with sufficient dispersion. In contrast to this passive method to deal with damage, the dispersion could be optimized from the start in order to minimize the chance of damage. The amount of damage should be determined quantitatively, regardless. With respect to the literature [36,38,62], a basic idea for the avoidance of scission of nano-particles during ultrasonic dispersion can be outlined. Lower power density by broader sound coupling (plate transducer instead of small horn, perhaps even a sonication bath) and lower power in general will be sufficient to break up agglomerates. At the same time, this reduction of overall power and power density will show rather stable cavitation, which is understood to reduce damage to the nano-particles. The lower power and the stable cavitation mode will thus reduce the risk of nano-particle scission and surface modification. Longer sonication times might be needed, but could be tolerable. At the same time, lower power density allows proper cooling of the containment, and reduces the amount of radical production by sonochemistry or damage to solvent systems.

## 4. Conclusions

Sonication is one of most promising routes for dispersion of CNT and other nanofillers like CNF into various liquid systems with low enough viscosity, like solvents or resin. While it is easily applicable and yields the possibility for a high degree of dispersion (i.e., small agglomerate size), it also potentially damages the liquid system by sonochemistry or the CNT by scission, surface damage, or both. The scission mechanisms, a lower length threshold reached after long sonication times, and the methods for determining length reduction quantitatively are well documented in the literature. Different parameters influence the probability for damage, dominated by the sonication power density and the overall deposited sonication energy. Others are the properties of the liquid; dimension of the sonicator, which again influences the power density; and the immersion depth of the horn. Many methods allow one to determine length reduction and surface integrity of the CNT, but also to quantify the dispersion quality. Thus, it seems feasible to apply quality control to a dispersion strategy and to optimize the process for optimal dispersion and minimal damage. This review gives an overview of relevant literature and will help future studies to access key information related to specific sonication strategies.

## Figures and Tables

**Table 1:** Overview of methods for monitoring damage and dispersion state.

Method	Application	Citation
TEM	CNT integrity, walls	[15,40,61,66,67]
SEM	Agglomerates, length, diameter,	[20,35,39,41,57,58,61,65,66]
AFM	Length diameter distribution	[25,36,48,64,68,69]
LM	Agglomerate size	[20,24,41,61]
Raman	Surface damage Amount of single CNT vs. bundles	[15,25,66,70,71] [84]
XPS	Chemical bonds, side groups, oxidation	[15,17,67,72,73]
UV–vis-NIR	Amount of single CNT vs. bundles	[25,35,36,38,48,69,75,76,77,86]
FTIR	Surface chemistry	[15,27,75]
DLS	Agglomerates and bundles size/indirect single CNT	[36,56,78]
XRD	CNT integrity	[72,74]
Rheology	Amount of well dispersed CNT	[24,61,79,83]
Conductivity	Dispersion state	[61,85]

**Table 2:** Overview of reported length reduction of CNT due to sonication, indicating the solvent system, the type of CNT, sonication power, and energy applied, as well as determined damage, together with the respective citation.

Dispersion Medium	Nanofiller Type	Input Power	Sonication Time/Energy Input	Analyzing Tool	Length Reduction	Measured Dimension	Citation
Distilled water + polyacrylate-based dispersing agent	MWCNT	150 W	25 J/mL–400 J/mL	SEM	265 nm (75 J/mL)–216 nm (400 J/mL); histogram peak	Length	[35]
Milli-Q water	Carboxylated MWCNT	100 W	7 h	SEM	902 nm (0 min)–152 nm	Length	[87]
Distilled water	MWCNT	260 W, ultrasonic bath	15–120 min	DLS	400 nm (0 min)–50 nm (90 min)	Length calculated from D_h_	[70]
D_2_O + sodium cholate	SWCNT	20 W	5–120 min	AFM	800 nm (0 min)–165 nm (120 min)	Length	[36]
Sodium deoxychlorate	SWCNT	120 W, sonication tip	10–120 min	AFM	1.3 µm (30 min)–0.7 µm (120 min) histogram peak	Length	[69]
Organic solvent + pyrene-siloxane surfactant	MWCNT	>60 W/cm^2^	3–10 h	SEM	5–15 µm (0 min)–2–6 µm (3 h)	Length	[65]
Sodium dodecyl sulfate + Milli-Q water	SWCNT (bundles)	20 and 40 W	15–120 min	DDLS	Length: 2.286–1.146 µm; diameter: 40.6–12.1 nm (20 W, 30–120 min); 1.156–0.822 µm; diameter: 37.9–2.9 nm (40 W, 15–120 min);	Length and diameter	[78]
Water	MWCNT and N-doped MWCNT	1.2 kJ, 2.1 kJ, 6 kJ, 10 kJ, 17 kJ	30–300 s	SEM	17.1± 11.9 µm undoped–13.6 ± 9.9 µm N-doped (30 s, 1.2 kJ); 9.5 ± 5.1 µm undoped–10.1 ±6.0 µm N-doped (300 s, 10 kJ)	Contour length	[67]

**Table 3:** Overview of the literature with respect to surface damage determined by Raman spectroscopy, including solvent system, type of CNT, input power, and sonication time, together with reported effect and the respective citation.

Dispersion Medium	Nanofiller Type	Input Power	Sonication Time/Energy Input	Analyzing Tool	Results	Citation
Distilled water	MWCNT	260 W, US bath	15–120 min	Raman	I_D_/I_G_ 0.96 (untreated)–1.2 (120 min)	[70]
NaNO_3_ + H_2_SO_4_ + KMnO_4_	MWCNT	/	40–160 min	Raman, FTIR	I_D_/I_G_ 0.84 (untreated)–1.14 (40 min); FTIR confirmed oxygen-containing functional groups	[15]
CH_2_Cl_2_	MWCNT	17 W	5–20 min	Raman, ESR spectroscopy	No info on ratio	[71]
o-DCB or DMF	SWCNT	195 W	20–220 s	Raman	I_D_/I_G_+ ratio increase by factor 1.4 (DMF) and 1.7 (o-DCB) after 60–80 s	[92]
ddH_2_O+distilled water+ss-DNA oligo+(dT)_30_	SWCNT	3–80 W, US bath	1–6 h	Raman	Almost no change, increase in defect sites less than 10% and not significant	[93]

**Table 4:** Overview of the literature with respect to monitoring the dispersion state of CNT, including information on the media into which CNT were dispersed, the CNT type, sonication power, energy input or sonication time, and characterization method applied, with a short summary of the results and the according citation for further reading.

Dispersion Medium	Nanofiller Type	Input Power	Sonication Time/Energy Input	Analyzing Tool	Results	Citation
Various surfactants (SDS, CTAB, polyxyethylene stearyl ether) and deionized water	MWCNT (neat and different functionalized types)	140 W	100 min	UV–vis	General finding for different surfactants and MWCNT: at short sonication time, absorbance increases rapidly, at higher times, absorbance levels off	[75]
NaDOC (30% cmc and 300% cmc) in Milli-Q water	SWCNT	100 and 200 W	0–60 min	UV–vis	100 W: ~0.775 (15 min)–0.88 (60 min) (30% cmc) ~0.825 (15 min)~1.25 (60 min) (300% cmc) 200 W: ~0.85 (15 min)–1.0 (60 min) (30% cmc) ~1.1 (15 min) ~1.95 (60 min) (300% cmc)	[38]
17 different surfactants	SWCNT	140 W	0–120 min	UV–vis	Resonance ratio increases: 0.04 (0 min)–0.08 (120 min) (surfactant: NaDDBS)	[86]
Distilled water + polyacrylate-based dispersing agent	MWCNT	150 W	25 J/mL–400 J/mL	UV–vis	Increase in absorbance: 0.4 (25 J/mL)–0.8 (400 J/mL)	[35]
Acetone	MWCNT	/(ultrasonic bath)	2–42 h	Rheology	Increasing viscosity values with increasing sonication time	[83]
Ethanol	MWCNT	/	2 h	Rheology	Shear thinning—viscosity values: ~106–~102 Pa.s (functionalized MWCNT); ~103–~101 Pa.s (neat MWCNT)	[80]
DCM	MWCNT	60–70 W	0–90 min	UV–vis + rheology	UV–vis: Increase of absorbance until 30 min sonication, afterwards decrease Rheology: After 30 min, network formation, afterwards, negatively affected	[61]

## Data Availability

Not applicable.
